# A Critical Review of the Removal of Radionuclides from Wastewater Employing Activated Carbon as an Adsorbent

**DOI:** 10.3390/ma15248818

**Published:** 2022-12-09

**Authors:** Anik Chakraborty, Animesh Pal, Bidyut Baran Saha

**Affiliations:** 1 Department of Nuclear Engineering, University of Dhaka, Dhaka 1000, Bangladesh; 2 International Institute for Carbon-Neutral Energy Research (WPI-I2CNER), Kyushu University, 744 Motooka, Nishi-ku, Fukuoka 819-0395, Japan

**Keywords:** adsorbents, activated carbon, biomass, radionuclide adsorption, removal techniques

## Abstract

Radionuclide-contaminated water is carcinogenic and poses numerous severe health risks and environmental dangers. The activated carbon (AC)-based adsorption technique has great potential for treating radionuclide-contaminated water due to its simple design, high efficiency, wide pH range, quickness, low cost and environmental friendliness. This critical review first provides a brief overview of the concerned radionuclides with their associated health hazards as well as different removal techniques and their efficacy of removing them. Following this overview, this study summarizes the surface characteristics and adsorption capabilities of AC derived from different biomass precursors. It compares the adsorption performance of AC to other adsorbents, such as zeolite, graphene, carbon nano-tubes and metal–organic frameworks. Furthermore, this study highlights the different factors that influence the physical characteristics of AC and adsorption capacity, including contact time, solution pH, initial concentration of radionuclides, the initial dosage of the adsorbent, and adsorption temperature. The theoretical models of adsorption isotherm and kinetics, along with their fitting parameter values for AC/radionuclide pairs, are also reviewed. Finally, the modification procedures of pristine AC, factors determining AC characteristics and the impact of modifying agents on the adsorption ability of AC are elucidated in this study; therefore, further research and development can be promoted for designing a highly efficient and practical adsorption-based radionuclide removal system.

## 1. Introduction

Water pollution with radionuclides has become a major environmental problem as the development of nuclear power has progressed. Usually, the contamination of water with radionuclides occurs either naturally or anthropogenically due to nuclear power plant accidents, leakage from a spent fuel pool and nuclear fuel processing facilities, or an act of nuclear terrorism [1]. Due to chemical and radioactive toxicity, exposure to radionuclide-contaminated water may pose more significant health risks, including cancer in many organs, birth defects, cognitive disorders and fertility problems [2]. Because radionuclides have a toxic nature and some of them have long half-lives, the World Health Organization (WHO, Geneva, Switzerland) and the United States Environmental Protection Agency (USEPA, Washington, DC, USA) have recommended maximum contamination levels of radionuclides in water [3]. As a result, numerous published research articles have focused on various techniques for removing radionuclides from aqueous solutions. Removing these radionuclides from water is very important for safe and healthy living.

Different removal techniques have been employed worldwide to remove radionuclides from water, such as adsorption [4], ion exchange [5], membrane filtration [6], evaporation [7], chemical precipitation [8] and electrodialysis [9]. Each method has its advantages and disadvantages. For instance, chemical precipitation is a technology that has several benefits, the most important of which are its cheaper installation cost and its ability to remove a majority of radionuclides. However, in addition to these benefits, there are also some downsides. Because this method generates a substantial quantity of sludges, the cost of sludge disposal is an additional factor of concern in this case [10,11]. The ion exchange technique comes with the advantages of clean effluent release and metal recovery features. However, along with these advantages, there are some drawbacks as well. The cost of installing an ion exchanger is relatively high, and not too many radionuclides can be removed using this technique [12,13]. The other mentioned techniques also have some pros and cons. Considering factors such as simplicity of design, ease of operation, high efficiency, a wider pH range and better removal of contaminants, even from dilute solutions, the popularity of adsorption as a removal technique is high worldwide, and this technique is more promising than the others [10].

In the adsorption technique, a solid material called an adsorbent is used to adsorb solute molecules from a liquid or gas. There are several adsorbents available for removing radionuclides, such as activated carbon [14], zeolite [15], graphene [16], carbon nanotubes [17] and metal–organic frameworks (MOFs) [18]. Each adsorbent has some advantages and drawbacks. For example, zeolite offers various benefits, such as reduced manufacturing costs, fewer equipment requirements and fewer area requirements. However, zeolite does not perform well in acidic water, and the regeneration process is challenging [19]. Metal–organic frameworks (MOF)s have excellent porosity and improved efficiency, but they also have drawbacks, such as longer reaction times, higher costs and increased byproduct formation. They are mainly in the research and development stage as of now [20,21]. The other listed adsorbents have certain advantages and disadvantages as well. Activated carbon is considered the most feasible adsorbent for wastewater treatment in terms of cost-effectiveness, availability, environmental friendliness, adaptability to the removal of various pollutants and adsorption performance [22]. Activated carbon can be produced from natural sources [21] or synthesized from other commercial carbon sources [23]. For example, Chinese liquor brewing wastewater (CLBW) is rich in ethanol and lactate, which can be treated as a valuable carbon resource [24]. Any materials derived from biomass are commended for their aforementioned qualities of abundance, renewability and low cost [25]. These low-cost locally available biomass/biomass wastes ensure the cost-effective manufacturing of activated carbon, and they also provide higher quality and enhanced adsorption performances [26]. Among biomass sources, rice husks [27], bamboo [28], sugarcane bagasse [29], potato peels [30], coconut shells [31], waste palm trunks [32,33,34] and mangrove woods [35,36,37] are rich in carbon content and hence can be used for the synthesis of activated carbon. These biomass sources are readily available in most countries, such as Bangladesh, and are also very effective for removing the concerned radionuclides. The adsorption performance of activated carbon can also be improved using different surface modification techniques.

According to literature reviews, it has been found that many researchers have used activated carbon for the removal of radionuclides from aqueous solutions. However, to the best of our knowledge, no significant comprehensive study has mentioned or compared removal technologies, the adsorbent performance of radionuclides adsorption and activated carbon modification techniques for performance enhancement. Therefore, first, this review demonstrates the harmful consequences of different concerned radionuclides. Then, it includes a comparative study of various removal techniques. This paper also conducts a comprehensive study on popular adsorbents frequently used to adsorb radionuclides. The surface properties of activated carbon and adsorption conditions, such as required temperature, pH level and observed concentration in different experiments, are studied in this paper. This study also provides ideas about the characteristics of activated carbon from other sources and indicates how efficiently they have performed practically in various adsorption environments. This review highlights why adsorption utilizing AC from biomass wastes is more viable than other radionuclide removal methods. It also summarizes the theoretical models used for correlating the isotherm and kinetics data of different radionuclide adsorption onto AC. Finally, this paper also discusses physical modifications, chemical modifications and variations in organic or inorganic loading techniques to modify the surface properties of AC to ensure better adsorption performance. 

## 2. Concerned Radionuclides with Associated Health Hazards

The release of energy from the nuclei of certain atoms and isotopes is referred to as radioactivity. Radioactivity can be detected in trace amounts in drinking water. The concentration and content of these radionuclides may vary with location due to the radiochemical constituents of the soil and rock layers through which the raw water may have passed. These radionuclides may have a natural or anthropogenic origin [38]. Naturally, radioactive elements are found in the earth’s crusts. There are also some anthropological radiation sources from which radioactive elements get mixed into the environment. Significant anthropogenic sources that have led to radioactive contamination of the environment are atmospheric nuclear weapons programs and testing [39], medical sources of radiation [40], industrial sources of radiation [41], nuclear fuel cycles of nuclear power plants [42], uranium mining and milling [43], geological deposits of high-level nuclear wastes that may emit radionuclides in the future, and last, nuclear accidents [44]. The majority of anthropogenic radionuclides are isotopes of cesium (^137^Cs), iodine (^131^I), strontium (^90^Sr), technetium (^99^Tc), plutonium (^239^Pu), cobalt (^60^Co), radium (^226^Ra) and radon (^222^Rn) [45]. When exposed to these radionuclides, they emit ionizing radiation, such as alpha, beta and gamma radiation, which can be harmful to the human body [46]. All three are ionizing radiation, which emits energy in the form of particles or waves with sufficient force to strip electrons from atoms [47]. In a technical report of IAEA (1973), it was stated that alpha particles can sometimes penetrate the skin’s dead layer. Inhaling radionuclides that alpha decay may result in a high dosage to the pulmonary epithelium [48]. According to a report by USNRC, although alpha particles have a limited range, they provide a far higher effective dosage than that of beta particles or gamma radiation [49]. Alpha particles are an environmental carcinogen due to their potent capacity to generate a high fraction of double-strand DNA breaks per particle. Repairing double-strand DNA breakage is more complex for the body [50]. Beta particles possess more penetrating power than alpha particles but far less capacity to ionize tissues and break DNA. Beta particles pose both internal and external radiation hazards. External beta radiation poses a more considerable risk than internal beta radiation [51]. However, Gamma rays, unlike alpha and beta rays, have no charge or mass. However, they are extremely penetrating radiation that may directly ionize atoms in the body and create secondary ionizations when their energy is transmitted to atomic particles, such as electrons. Gamma rays provide a more considerable external radiation hazard [50].

The energy deposited by alpha, beta and gamma radiation may cause cell damage. The effect of radiation on a cell depends on the time of exposure, the dose rate, the total quantity of energy absorbed and the exposed tissue or organ. The probability of cell damage rises as the dosage increases [48]. In 2011, Tawn et al. [52] and Bunin et al. [53] explained that, although radiation-induced heritable mutations have not been documented in the children of Japanese atomic bomb survivors, there is some evidence to indicate that radiation-induced heritable mutation can be found in humans. Cesium, cobalt, strontium, uranium and iodine radionuclides emit ionizing radiation and create various health effects. This review lists some concerning radionuclides and their associated health hazards, as shown in Table 1. For example, a report by the CDC [52] stated that excessive Cs-137 exposure can result in burns, serious radiation illness and even death. Because of exposure to high-energy gamma radiation, Cs-137 may raise the risk of cancer. Internal Cs-137 exposure by ingestion or inhalation causes the radioactive material to be dispersed in soft tissues, particularly muscle tissue, which exposes these tissues to beta particles and gamma radiation and increases cancer risk. In another report by the USNRC, the beta dose rate from uranium decay products is minimal immediately after uranium separation. However, it may provide a beta dose rate with the contact of around 150 mrem/hr many months later due to the accumulation of ^234^Th [50]. The Agency for Toxic Substances and Disease Registry (ATSDR) recently issued a comprehensive analysis of the adverse health consequences caused by uranium. This case study concluded that renal toxicity caused by high doses of uranium can result in death [54]. The primary objective of this review is to study the removal of these radionuclides from the water that may pose health problems if they are not eliminated. Table 1 summarizes some of the most significant characteristics of those radionuclides and their associated health risks.

Some concerning radionuclides are mentioned in the above table. Among these radionuclides, zinc-65 and radon have comparatively lower half-lives. Therefore, these two radionuclides are not considered in the latter part of this study. The other nuclides, such as cesium-137, strontium-90, cobalt-60, uranium and iodine-131, significantly impact human health and the environment, as these radionuclides have long half-lives (see Table 1), are abundant in nature and are also more commonly released from nuclear facilities in normal and accidental situations [67]. For instance, during the Three Mile Island nuclear accident in 1979, fission products that vented into the atmosphere were ^137^Cs, ^131^I and ^85^Kr [68]. The next big nuclear disaster happened in 1986 in Chernobyl. This is regarded as the most devastating accident in the history of the nuclear power industry. Iodine-131, cesium-134 and cesium-137 were the primary radionuclides discharged from the reactor and were responsible for exposing radiation [69]. The Fukushima Daichi nuclear disaster was another significant event that happened recently in 2011. According to the Japanese government’s newest report [70], a total of sixty-two (62) radioactive isotopes were discovered in Fukushima’s existing nuclear water tanks, with the quantity of a radioactive isotope called tritium reaching around 860 TBq^2^. A survey found that the most significant amounts of released radionuclides in the Fukushima accident were cesium, strontium, iodine and cobalt [70]. These radionuclides are also often found in waste generated from industrial and medical radioisotope applications [40]. That is why further studies that we mentioned in this paper focus on these radionuclides.

## 3. Different Removal Techniques

**Figure 1:**
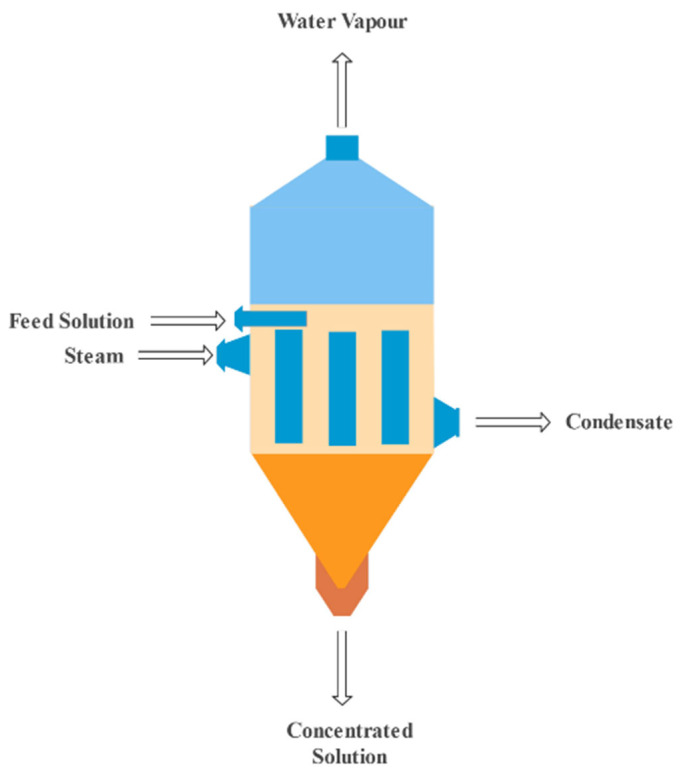
Evaporation technique [71].

Removal techniques refer to the procedures used to remove radionuclides from water. Various techniques have been studied. The processes may include adsorption, evaporation, ion exchange, electrodialysis, membrane filtration and chemical precipitation. Evaporation is a method for recovering valuable items or isolating harmful compounds from wastewater flows. By vaporizing a part of the solvent, evaporation concentrates a solution. This technique is excellent in concentrating or removing salts, heavy metals and other dangerous substances from waste effluent. In 2005, Kim et al. [7] studied the treatment of organic radioactive wastes using the evaporation method. The evaporation approach was used in this research to dispose of large organic wastes, such as solvents used in equipment cleaning and extraction processes. The study discusses the properties of those wastes, the influence of temperature and solvent concentration on evaporation and the time required for complete evaporation using pure solvents. Figure 1 demonstrates a schematic representation of a typical evaporation technique.

**Figure 2:**
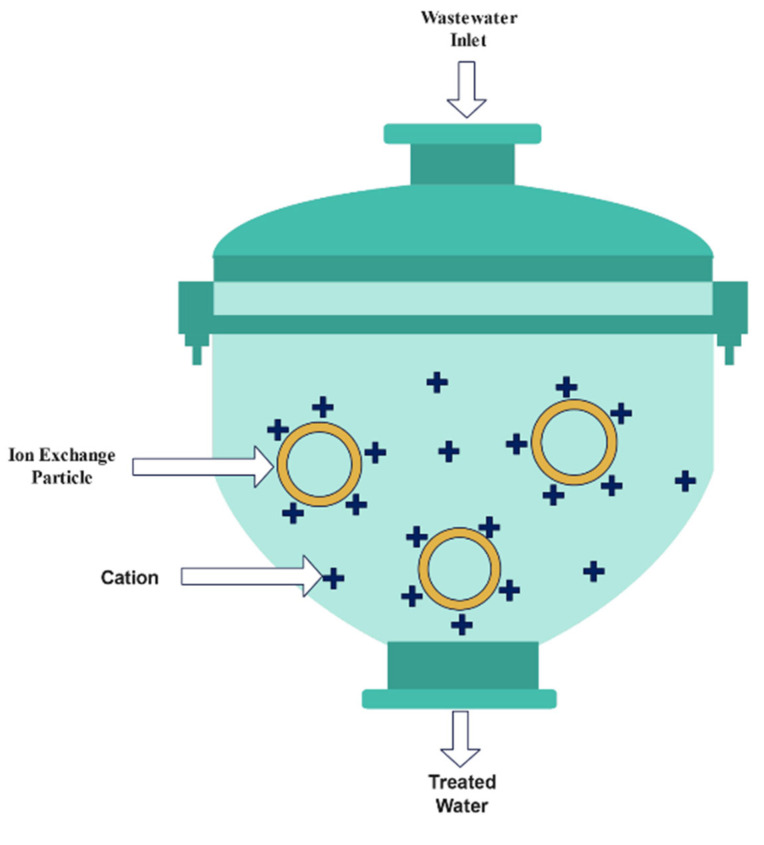
Ion exchange technique [74].

Ion exchange is a water purification technique in which one or more undesired ionic impurities are exchanged for a non-objectionable ionic material. In 2016, Yang et al. [72] removed cesium-137 using an ion exchange method based on zeolitic chalcogenides (RWY) that was effectively applied. K@RWY could promptly capture Cs+ with excellent selectivity, high capacity, good acids and alkali, and superior radiation resistance. The adsorption capacity of K@RWY was 298 cm^3^ g^−1^. K@RWY was shown to be an exceptionally promising ion exchanger for removing radioactive Cs, according to the results. Canner et al. [5] employed Purolite S930+, Purolite S950, Purolite S957 and Dowex M4195 resins to remove some radionuclides from an HCl steel decontamination stream to understand the extraction mechanisms better and to identify a potential steel decontamination process that could help reduce the volume of intermediate-level waste generated during nuclear reactor decommissioning. A two-stage technique was proposed for this investigation: a radionuclide strip using chelating resins, such as Purolite S930+ or a Purolite S950 column, followed by another chelating resin, a Dowex M4195 column. Marinin and Brown [73] studied sorbent/ion exchange materials to remove radioactive strontium from liquid radioactive waste and high-hardness groundwaters. The Amberlite IRC-718 and Duolite C-467 samples demonstrated the most excellent distribution coefficients [73]. Figure 2 shows a visual representation of a typical ion exchange technique.

**Figure 3:**
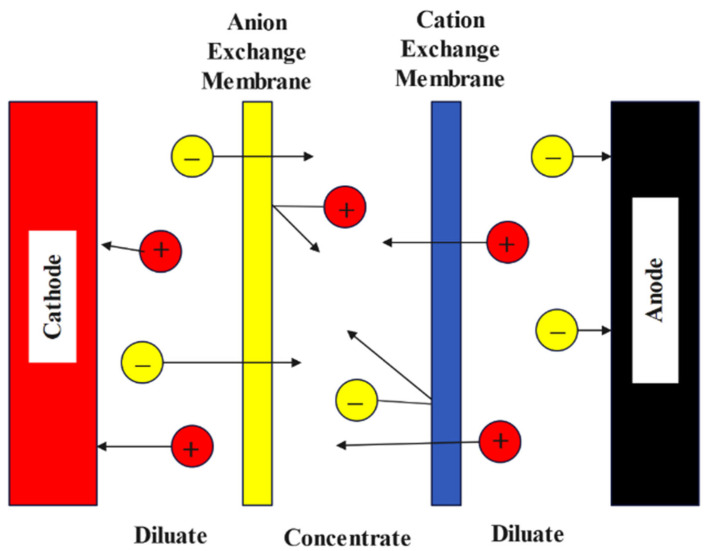
Electrodialysis technique [78].

Electrodialysis (ED) is a technique for removing ions from an aqueous solution with electrically charged membranes and an electrical driving potential [75]. It is exclusively effective on ionizable substances. Miskiewicz et al. [9] conducted research in 2021 on the possible application of electrodialysis for the treatment of low-level radioactive liquid waste, namely for the removal of cesium and cobalt, which was the primary emphasis of their study. Electrodialysis is a good approach for treating radioactive liquid waste because it provides high desalination rates and enough levels of radioactive material removal. This research demonstrated that ED can be used to separate both organic and inorganic pollutants. In 2019, Alkhadra et al. [76] studied the continuing separation of radionuclides from polluted water using shock electrodialysis. This study focused on the underlying physics and design ideas necessary to selectively extract cobalt and cesium while recovering a considerable amount of the supplied water and minimizing the energy cost of the extraction process. Around 99.5% of cobalt can be extracted with a 43% water recovery rate, and up to 66% of water can be recovered if cobalt deionization can be reduced to 98.3%. In 2014, Zahakifar et al. [77] examined strontium removal from aqueous solutions using electrodialysis (ED). Maximum removal efficiency (99.4%) was achieved under working circumstances with a flow rate of 5 mL min^−1^ and a voltage of 30 V. According to the findings, boosting voltage and reducing flow rate enhance performance. A schematic diagram of a typical electrodialysis technique is shown in Figure 3.

**Figure 4:**
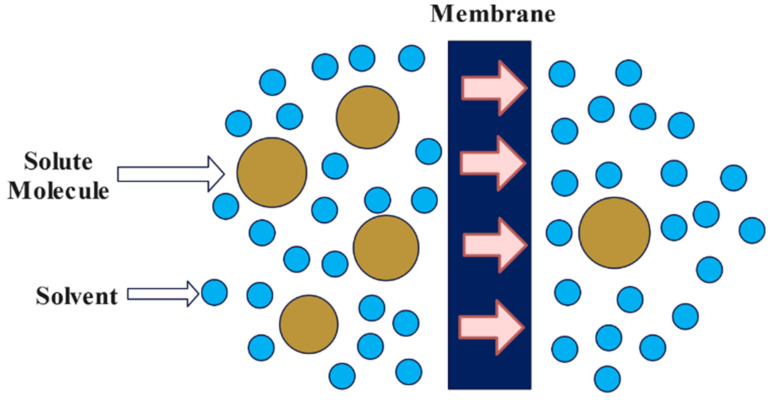
Membrane filtration technique [81].

Membrane filtration (MF) is a pressure-driven separation process that employs a membrane to sieve particles and macromolecules mechanically and chemically. Clifford et al. [6] examined three unique reverse osmosis (RO) modules: thin-film polyamide hollow fiber, a low-pressure composite spiral wound and a thin-film composite. For the regular pressure modules (the first and third modules), 226-Ra rejection exceeded 99%; however, it was only 91% for the low-pressure module. In each of the three situations, radium rejection was somewhat larger than hardness rejection, showing that hardness monitoring can be used instead of radium monitoring. According to Havener et al. [79], membrane filtering methods eliminate 87–98% of radium in potable water. The activity of alpha particles, beta particles and photon emitters may all be removed similarly. In 2015, Ding et al. [80] studied cesium (Cs) and strontium (Sr) removal from synthetic water containing Suwannee River organic matter, natural surface water (SW) and a wastewater effluent (WW) using two types of ultra-low pressure composite polyamide RO membranes: RE1812-50 (M1, Republic of Korea) and TW30-1812-50 (Beijing, China) (M2, Minneapolis, MN, USA). The Sr rejections by membranes M1 and M2 approached 97.5 and 96%, respectively, whereas Cs rejections were above 90% and 85%, respectively. In Figure 4, a visual representation of a typical membrane filtration technique is demonstrated.

**Figure 5:**
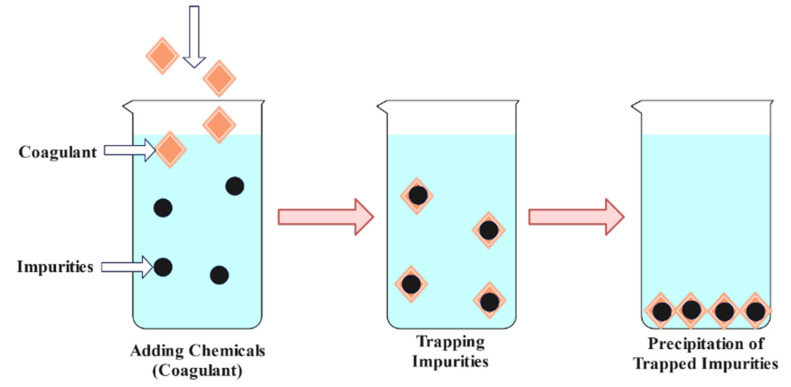
Chemical precipitation technique [84].

Chemical precipitation transforms a solution into a solid by converting the solute into being insoluble or by supersaturating the solution. Mollah et al. [82] investigated the elimination of Cs-134 and Co-60 from liquid radioactive waste generated using the chemical precipitation method. Using an ideal nickel concentration of 0.75 M and an optimal cyanide concentration of 0.50 M, approximately 98% of Cs-134 can be eliminated at an optimal pH of nearly 10. Using the same technique, over 60% of Co-60 can be eliminated. Bovrob et al. [83] examined the viability of removing artificial radionuclides from drainage water and groundwater by chemical precipitation and sorption procedures. Before phosphate precipitation, they analyzed the two-step treatment procedure, which included sodium carbonate softening or phosphate precipitation. In a 2018 study, Ahmet E. Osmanlioglu [8] presented two-stage chemical precipitation to filter radioactive wastewater using chemical agents. Principal radionuclides included Cs-137, Cs-134 and Co-60. At high pH levels of 8–10, potassium ferrocyanide, nickel nitrate and Ferrum nitrate were used as chemical agents. A visual representation of a typical chemical precipitation technique is shown in Figure 5.

**Figure 6:**
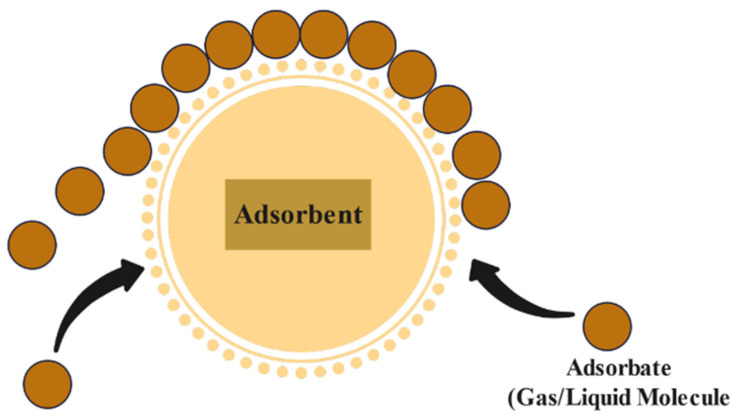
Adsorption technique [85].

The process of transferring molecules from a fluid bulk to a solid surface is known as adsorption, as shown in Figure 6. Adsorption is a surface process that can result from chemical bonding or the action of physical forces. This method can be broken down into two distinct parts. The term “adsorbate” refers to the molecular species that is adsorbed on a surface, whereas “adsorbent” describes the surface where the adsorption process occurs [4]. Several research articles on removing radionuclides by the adsorption process are mentioned later in this paper. 

Every removal technique has different working processes. They are quite different processes, and their performances are also different. Table 2 below lists the benefits and limitations of various removal strategies.

Each removal method has its own set of pros and cons. However, adsorption is the most popular and cost-effective removal method that has few drawbacks and is still mostly used [89]. The adsorption performance of radionuclides largely depends on the surface, physical and chemical properties of the adsorbents. Therefore, the following section studies the performance of different adsorbents and adsorption conditions.

## 4. Performance Comparison of Different Adsorbents

Adsorption is a physical process in which atoms, ions, or molecules attach to a surface from a gas, liquid or dissolved solids. This may occur from gases, liquids or solids that have been dissolved. As a consequence of this, an adsorbate layer is produced on the surface of the adsorbent. Both van der Waals forces and interactions based on hydrophobic repulsion are responsible for the attraction. The method’s objective is the removal of contaminants or the recovery of valuable components from a polluted supply stream. Activated carbon, zeolite, graphene, nanotubes and MOFs are some of the most widely used adsorbents. Each of these adsorbents is synthesized differently, and they also vary in terms of performance and properties.

Activated carbon (AC) is a widely used solid adsorbent material. Chen et al. [14] demonstrated the use of activated carbon generated from rice husks and assembled a comprehensive bibliography and future outlooks on rice husk activated carbon. Zeolite is an aluminum silicate that occurs naturally but may also be manufactured. Each zeolite variant has same-sized pores throughout its crystal structure. Frederick A. Mumpton discussed the key features of natural zeolites and their significant applications in pollution management, nuclear waste processing and storage, agriculture and biotechnology [15]. Graphene, a two-dimensional allotrope of carbon made of carbon atoms arranged in a honeycomb lattice, is another adsorbent that has recently come into usage. Sophia et al. [16] examined graphene materials in pharmaceutical wastewater treatment and described the adsorption process. Carbon nanotubes (CNTs) are cylindrical molecules made of single-layer carbon atom sheets. SWCNTs (single-walled carbon nanotubes) have a diameter of less than a nanometer, but MWCNTs have a diameter of more than 100 nm. They are made of multiple concentrically connected nanotubes. Ihsanullah et al. [17] systematically evaluated the use of carbon nanotubes to remove heavy metals from water. They also discussed the operational factors, processes, present roadblocks and future implications of technology. In the field of adsorbents, metal–organic frameworks (MOFs) are a new addition still in the research phase. MOFs are porous inorganic–organic materials with a metal–oxygen cluster. In a publication, Camille Petit outlined current research developments on this topic, including novel adsorption uses, manufacturing and the significance of modeling. In addition, she highlighted the most promising structures for specific applications, such as molecular separation, water purification and harvesting, and she provided a vision for future studies in this sector [18]. Table 3 illustrates those adsorbents’ benefits and disadvantages. 

Each adsorbent mentioned above has its distinct function and activation mechanism. Adsorption performances and manufacturing costs of adsorbents differ because of the varied methods employed to fabricate their structures. Among the adsorbents, activated carbon is a potential and cost-effective candidate [95]. Many researchers have studied how these different adsorbents perform in adsorbing different radionuclides from solutions. The most important aspect of their research is demonstrating these adsorbents’ maximum adsorption capacities and removal efficiencies. Temperature, pressure, pH level and starting concentration are all significant factors in these investigations because these adsorbents exhibit maximal effectiveness in certain conditions. Khandaker et al. [96] examined the adsorption of cesium (Cs) from an aqueous solution using air-oxidized bamboo charcoal (BC). They found that the adsorption capacity and removal efficiency are 55.25 mg/g and 98%, respectively. The study was conducted in a temperature range of 288–308 K, and the pH level was 2–12. In another study in 2019, Teng et al. [97] studied uranium (VI) adsorption from an aqueous solution with a modified rice stem. They found that the adsorption capacity and removal efficiency are 11.36 mg/g and 97.6%, respectively. This maximum adsorption capacity was found at the temperature of 298 K, and the pH level was 4.

Similarly, in 2019, Eljamal et al. [98] synthesized novel nanoscale zero-valent iron–zeolite (nZVI–Z) and nano-Fe/Cu–zeolite (nFe/Cu–Z) composites for cesium removal. They found that the adsorption capacity and removal efficiency are 77.51 mg/g and 73.72%, respectively, for nFe, and they are 71.12 mg/g and 63.11%, respectively, for nZVI–Z. The maximum adsorption capacity was found in both cases when pH levels were 4. In 2010, Yavari et al. [99] studied the sorption of strontium ions from aqueous solutions with oxidized multiwall carbon nanotubes. They found that the adsorption capacity and removal efficiency are 6.62 mg/g and 85%, respectively. The maximum adsorption capacity was found at 298 K, and the pH level was 6.5. Table 4 summarizes different research that shows the adsorption capacities and removal efficiencies of various adsorbents (i.e., activated carbon, zeolite, graphene, CNT and MOF) in removing various radionuclides (i.e., cesium, strontium, iodine, uranium and cobalt). The table also demonstrates the optimum temperature, pressure, pH range and initial concentration of adsorbates for the maximum adsorption capacities and removal efficiencies achieved in those studies.

Table 4 demonstrates that the adsorption capacities of radionuclides onto activated carbon are very high. Additionally, the process can be more cost-effective and ecologically favorable by producing activated carbon from widely available biomass sources. Consequently, in the following sections, this study focuses on activated carbon as an adsorbent for the adsorption of radionuclides.

## 5. Synthesis Process of Activated Carbon and Influential Factors

**Figure 7:**
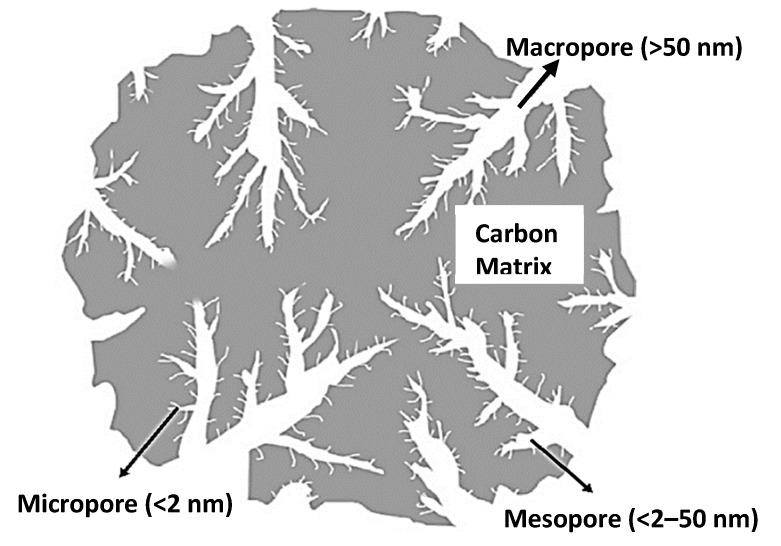
Porous structure of activated carbon [169].

In the broadest sense, activated carbon (AC) refers to an amorphous carbonaceous material characterized by a high degree of porosity and a sizeable inter-particle surface area. It is widely used as an adsorbent. There are different types of activated carbon, such as granular (GAC), powdered (PAC), impregnated (IAC), extruded (EAC), bead-type (BAC), etc. [165]. The most used type is powdered activated carbon. Similar to other adsorbents, the surface area, porosity, pore volume, adsorption capacity, chemical inertness and stability are the main deciding properties for the better performance of activated carbon [166]. Previously, most organic resources could be employed as precursors for producing activated carbon. Today, biomass precursors are utilized more frequently due to their low cost, availability and renewability. Understanding the relationship between activated carbon’s porous characteristics, including surface area, pore size distribution and adsorption capacity, are vital for selecting the most suitable adsorbent for the efficient removal of radionuclides. Highly developed micro- and mesopore structures are a defining characteristic of porous carbons. For the adsorption of smaller molecules, micropores are necessary [167]. The International Union of Pure and Applied Chemistry (IUPAC) has defined three groups of porous activated carbon according to pore size: macropores (>50 nm diameter), mesopores (2–50 nm diameter) and micropores (<2 nm diameter). Each of the three pore sizes of activated carbon has a distinct purpose. Some pores which have diameter less than 0.7 nm also exist in activated carbon matrix. These pores are called ultra-micropores. Micropores are the most essential pores because of their large surface area, which provides them with a greater adsorption capacity due to their smaller size. This is comparable with molecules that are adsorbed in micropores. Molecules with sizes ranging between those of micropores and macropores are often retained in mesopores. Macropores are pores that are too large to be filled by capillary condensation. The primary purpose of macropores is to expedite the transport of the adsorbates to smaller pores placed deeper inside the activated carbon. However, macropores may also hold large molecules, such as humic acids, formed during the decomposition of organic matter [168]. Figure 7 shows the hierarchical porous structure of activated carbon.

Carbon is mainly activated through two processes. One is physical activation, and the other is chemical activation. Carbon can be physically activated using high-temperature steam [170] or by using both CO_2_ and steam [171]. The Boudouard reaction is involved in the activation process with CO_2_ [172]. The equations are as follows:

*C_f_ + CO_2_→C(O) + CO_2_…………………. [Chemisorption by dissociation]*

*C(O)→CO………………………………. [Surface oxide desorption]*

The overall reaction can be written as follows [173]:

*C(s) →CO_2_(g)→2CO(g);* Δ*H = +159 kJ mol^−1^*

The smaller size of water molecules relative to CO_2_ molecules makes activation with steam easier. Because the reaction is endothermic, it is simpler to manage. Sajjadi et al. [172] and Lussier et al. [173] described the interactions between carbon and steam. The equations are as follows:

*C_f_ + H_2_O→C(O) + H_2_……………………………[Chemisorption]*

*C(O)→CO + C_f_…………………………………. [Scavenging of surface oxide]*

*CO(g) + C(O)→CO_2_(g) + C_f_…………………… [Carbon gasification]*

*CO + H_2_O→CO_2_ + H_2_…………………………… [Water-gas shift reaction]*

*C_u_ + 2H_2_O→CO_2_ + 2H_2_………………………… [Carbon gasification by steam]*

*C_f_ + CO_2_→2CO………………………………… [Carbon gasification by carbon dioxide]*

*C_f_ + 2H_2_→CH_4_……………………………… [Carbon gasification by hydrogen]*

*CH_4_ + H_2_O→CO + 3H_2_……………………… [Carbon gasification by hydrogen]*

The overall reaction can be written as [174]

*C(s) →H_2_O(g)→CO(g) + H_2_(g);* Δ*H = +117 kJ mol^−1^*

Activated carbon can also undergo the chemical activation process using different chemical activating agents, such as NaOH [175], KOH [176], H_2_SO_4_ [177], ZnCl_2_ [22], H_3_PO_4_ [178], etc. For example, potassium hydroxide (KOH), despite its basic character, is often used to induce porosity through solid–solid or solid–liquid processes [179]. The reactions between KOH and carbon are as follows [180]:

*2KOH→K_2_O + H_2_O………………………. [Dehydration]*

*C + H_2_O→H_2_ + CO………………………… [Water-gas reaction]*

*CO + H_2_O→CO_2_ + H_2_ ………………….…. [Water-gas shift reaction]*

*K_2_O + CO_2_→K_2_CO_3_ …………………….…. [Carbonate formation]*

*C + K_2_O→2K + CO …………………….…. [Reduction by carbon]*

*6KOH + 2C→2K + 3H_2_ + 2K_2_CO_3_ ………. [Global reaction]*

*K_2_CO_3_ →K_2_O + CO_2_*

*CO_2_ + C→2CO*

*K_2_CO_3_ + 2C→2K + 3CO …………………. [Reduction by carbon]*

*K_2_O + H_2_→2K + H_2_O ………………………. [Reduction by hydrogen]*

**Figure 8:**
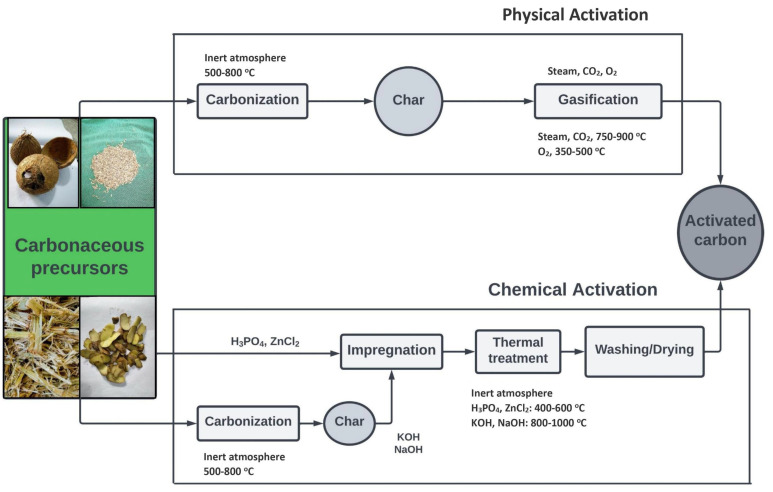
Physical activation and chemical activation flow chart [181].

The general steps of both physical and chemical activation processes are shown in a flow chart in Figure 8.

In most cases, chemical activation is preferred over physical activation due to higher yields (27–47 wt%) than those of physical activation (6 wt%), and due to minor surface damage to the produced activated fibers [182]. Moreover, chemical activation has a lower activation temperature, shorter activation period, and more remarkable porosity development [183].

**Figure 9:**
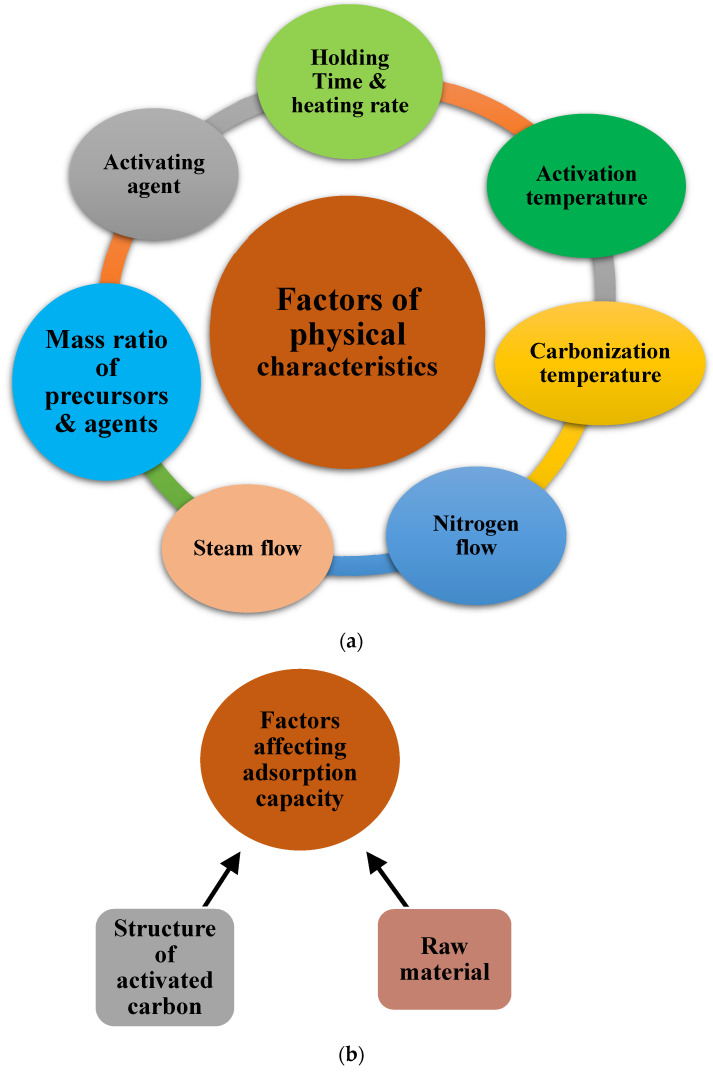
Factors influencing the (**a**) physical characteristics and (**b**) adsorption capacity.

The crucial factors that substantially affect activated carbon’s physical characteristics are as follows and schematically represent in Figure 9a:

- **Activating agents:** Activating agents play significant roles in the activation process, and various utilized chemicals react differently depending on the type of biomass and the employed temperatures. Under high thermal conditions, the reactivity of chemical activating agents with biomass materials produces an intermolecular reaction that results in efficient AC production. Numerous activating chemicals have been widely used to produce activated carbon with the required pore structure. The objective of the activation process is to generate and expand (volume and size) porosity in the carbon material, enhancing its adsorptive ability. To produce activated carbon, the lignocellulosic precursor is mainly impregnated or physically mixed with a chemical agent, such as H_3_PO_4_, H_2_SO_4_, HNO_3_, NaOH, KOH or ZnCl_2_ [181].
- **Holding time and heating speed:** The holding time significantly impacts activated carbon’s removal efficiency and adsorption capacity. In 2017, Sun et al. [184] found that metronidazole elimination efficiency is 91% after 60 min, climbing to 98% at 120 min and remaining stable. According to the findings published by Shaaban et al. [185], a more extended holding period leads to the creation of well-defined pores in biochar and an increase in BET surface area. During pyrolysis, rapid heating generates macroporous residue. Low-speed heating ramps are often used to prepare activated carbon. This method permits the complete combustion of material precursors and enhances the development of porosity [186].
- **Activation temperature:** The activation temperature substantially affects the pore structure and adsorption properties of AC. Lan et al. [187] discovered that, when activation temperature increases, the iodine adsorption value first climbs and then falls, and the yield continuously decreases. The best temperature range for the activation procedure is 900 °C to 1000 °C. The highest values for specific surface area and pore volume are 636.91 m^2^ g^−1^ and 0.363 cm^3^ g^−1^, respectively.
- **Carbonization temperature:** The influence of carbonization temperature on activated carbon removal efficiency and adsorption capacity is substantial. Osman Unera and Yuksel Bayrak [188] found that, when the carbonization temperature is raised from 300 to 400 °C, the AC surface areas increase; however, the AC surface areas again decrease at over 400 °C. Carbonization temperature and time significantly impact the pore structure of activated carbon. It is connected to the increased density of activated carbon [189].
- **Nitrogen flow:** The adsorption characteristics are interestingly affected by gas flow, especially nitrogen flow. In 2007, Stavropoulos et al. [190] found that adding nitrogen to activated carbon promotes the formation of a microporous structure. Thermal treatment of activated carbon in a urea-saturated gas flow mainly produces microporous samples with large pore volumes. Nitrogen functionality enhances the phenol adsorption capability of raw activated carbon.
- **Steam flow:** Steam flow is most considerable in the physical activation method. In 2018, Bergna et al. [191] showed that the steam flow rate dramatically impacts the yield and the total carbon and oxygen contents. Steam flow and holding duration have similar effects on pore size distribution, with increased responses and a greater impact on mesopore production (0.054 to 0.156 cm^3^ g^−1^).
- **Mass ratio of precursors and activating agents:** This results in less chemical agent use and better excess removal during the carbon washing process. The impact of increasing the fraction of impregnation over the porous carbon structure is more significant than that of increasing the carbonizing temperature [192].

Some factors affect the adsorption capacity of activated carbon. These are as follows and schematically represent in Figure 9b:

- **Raw materials:** Activated carbon is made from various carbonaceous compounds derived from animals, plants, minerals, anthracite, petroleum coke, coal and lignocellulosic waste products, such as wood, walnut shells, coconuts or almonds [193]. Although any carbonaceous substance may be suited to be a promising adsorbent, it must fulfill certain criteria to be utilized commercially. These requirements include availability, cost and the production of compatible activated carbon for all applications. The material’s composition determines the quality of the adsorbent.
- **Structure of activated carbon:** The adsorption capacity of activated carbon is strongly influenced by its structures, such as its porous structure, crystalline structure and chemical structure [194]. The activation procedure eliminates disordered carbon by exposing the crystallites to the activating chemical, forming a porous structure. According to Prahas et al. [195], the high adsorptive capacities of activated carbon are strongly correlated with porous properties such as surface area, pore volume and pore size distribution.

Activated carbon can be derived from different sources. Kawano et al. [196] prepared activated carbon from petroleum coke with KOH activation. It can also be prepared from carbonaceous biomass sources. The process becomes more economical and environmentally friendly if activated carbon is manufactured from abundant biomass sources. Activated carbon produced from biomass precursors can be a potential adsorbent to adsorb radionuclides from water.

## 6. Potential Biomass Sources for Activated Carbon Production

Activated carbon can be produced using various precursors, including biomass and non-biomass. Precursors range significantly in their structure and carbon content. Exploiting biomass as a precursor for making AC can be deemed a wise approach because the process is environmentally benign; the precursor is abundant and has a low cost compared with non-renewable sources. This research aims to identify viable biomass precursors that can be used to prepare high-quality activated carbon which has high potential for the adsorption of radionuclides. The most abundant precursors that can be used for the production of activated carbon are rice husks, bamboo, coconut shells, potato peels, sugarcane bagasse, waste palm trunks and mangrove wood [197,198,199]. Table 5 shows the favorable weather conditions, annual production and availability of the above-mentioned biomass precursors produced in Bangladesh as an example.

The procedure for manufacturing activated carbon from these various biomass sources differs in some respects. There are differences in the activation techniques and in the activating chemical often employed to activate carbon. In 2016, Hariprasad et al. [206] studied the characteristics of activated carbon produced from rice husks. They used KOH as the activating agent, and the activation temperature was 600 °C. They outlined the complete activation procedure in their article. In another study in 2013, Song et al. [207] studied the activated carbon of coconut shells for the removal of Pb from aqueous solutions. They used KOH as an activating agent, and 500 to 700 °C was the activation temperature range. Some of the activation conditions from various biomass sources are summarized in Table 6.

## 7. Surface Properties and Adsorption Capacity of Biomass-Derived AC

Assigning several essential criteria and emphasizing several essential traits is necessary to achieve optimal adsorption. There are three important parameters to consider: pore size, surface area and pore volume. Activated carbon performs better for adsorption if these parameters are within acceptable ranges. Yalcin and Sevinc [27] measured the surface area and porosity of activated carbon made from rice husks in 2000 m^2^ g^−1^. Utilizing ZnCl_2_ as the activating agent, the surface area, pore volume and pore size were determined to be 480 m^2^ g^−1^, 1.365 cm^3^ g^−1^ and 4.4 nm, respectively. Tran et al. [218] assessed the surface area and porosity values of sugarcane bagasse-activated carbon. Using ZnCl_2_ as the activating agent, the surface area, pore volume and pore size were determined to be 1495 m^2^ g^−1^, 0.88 cm^3^ g^−1^ and 0.85 nm, respectively. Table 7 outlines the key surface characteristics of various biomass-derived activated carbon.

After studying surface properties, it is important to observe in what conditions these precursors show the highest adsorption capacity. Olakunle et al. [227] investigated the development of AC from rice husks for cesium removal. They found that this AC shows a maximum adsorption capacity of 13.58 mg g^−1^ when the activation temperature, pH level and initial adsorbate concentration are 400 °C, pH 6 and 70 mg L^−1^, respectively. In 2018, Hayakawa et al. [28] studied strontium removal using bamboo-based activated carbon. AC showed a maximum adsorption capacity of 32.62 mg g^−1^ when the activation temperature, pH level and initial adsorbate concentration were 800 °C, pH 9.5 and 10 mg/L, respectively. Kyzas et al. [228] investigated the production of activated carbon derived from potato peel waste to remove cobalt ions. They found that these adsorbents show a maximum adsorption capacity of 405 mg g^−1^ when the activation temperature, pH level and initial adsorbate concentration are 600 °C, pH 6 and 200 mg L^−1,^ respectively. They used H_3_PO_4_ as the activating agent. Table 8 shows how different biomass-precursor-derived AC performs in adsorbing radionuclides from aqueous solutions. The pH levels and adsorbate concentrations in which these precursors show their maximum adsorption capacities are listed in this table.

## 8. Adsorption Isotherm Models

The adsorption isotherm is essential for distinguishing the distribution of adsorbate molecules between the liquid and solid phases on the adsorbent surface at equilibrium and the adsorbing properties of an adsorption system. The findings can also be used to compare the performance of adsorbents in terms of their adsorption capacities for various pollutants [246]. Adsorption isotherms often explain the relationship between the two parameters, *q_e_* and *C_e_*, under constant temperature and pH in terms of how various adsorbates interact with an adsorbent. Information obtained from an isotherm is currently used to design efficient and financially viable commercial treatment systems, as well as to learn more about adsorbent capacities, the adsorption phenomenon, the expression of surface properties and the optimization of adsorption mechanism pathways. Numerous equilibrium isotherm models have been used throughout the years to describe radionuclide adsorption onto activated carbon. Some of the most used isotherm models include Langmuir, Freundlich, Dubinin–Radushkevich and Temkin models [247]. Among these models, adsorption isotherms are primarily expressed in terms of the Langmuir and Freundlich isotherms [248].

### 8.1. Langmuir Model

The Langmuir adsorption isotherm was first established to explain gas-to-solid-phase adsorptions, but it was later used for the solid–liquid interface [249]. The surface covering is given as a fractional coverage and is dependent on the adsorbate concentration. The mathematical development is based on the physical simplicity of the processes regarding the following alternative hypotheses: (1) the surface is homogenous, meaning all sites are energetically analogous; (2) adsorption is a monolayer process, and each site may only adsorb one adsorbate molecule; (3) there is no lateral contact between adsorbed molecules; and (4) adsorption is reversible. The Langmuir isotherm model is likely one of the most well-known and commonly used models for fitting a vast array of experimental isotherm data [249]. This model can be expressed as

$$\frac{q_{e}}{q_{m}}=\frac{K_{L}Ce}{1+K_{L}Ce}$$

where *q_e_* represents instantaneous removal (mg/g), *q_m_* represents maximum removal (mg/g), *C_e_* represents the equilibrium concentration (mg/L) and *K_L_* represents the Langmuir constant (L/mg) [250].

### 8.2. Freundlich Model

Unlike the Langmuir isotherm, this empirical model may be used for multilayer adsorption at heterogeneous sites. It assumes that the adsorption heat distribution and surface affinities are not homogeneous. Energy distribution for adsorptive sites presents a spectrum of diverse binding energies as opposed to a single uniform energy and follows an exponential function, which is similar to the actual situation [251]. The Freundlich isotherm represents multilayer adsorption under the assumption that the energy distribution of adsorbed sites decays exponentially. Nevertheless, it does not apply to various adsorption data [252]. This model can be expressed as

$$q_{e}=\left(K_{f}\right)\left(C_{e}^{\frac{1}{n}}\right)$$

where *q_e_* represents the adsorbate removal (mg/g), K_f_ represents the Freundlich constant (mg/g) (mg/L)^−(1/*n*)^ and 1/*n* represents the heterogeneity factor. If *n* = 1, Freundlich’s model becomes Henry’s law [252].

### 8.3. Temkin Model

The Temkin model, which assumes a multilayer adsorption process, take into account interactions between the adsorbent and the adsorbate but ignores very low and extremely high concentration values. The Temkin model can be expressed as

$$q_{e}=\frac{RT}{b}lnC_{e}+\frac{RT}{b}lnK_{m}$$

where *R* represents the universal gas constant in J/(mol.K), *T* represents the temperature in *K, b* represents the Temkin constant associated with the sorption heat in J/mol and *K_m_* represents the Temkin isotherm constant in L/g. The result of plotting *q_e_* against ln *C_e_* is a straight line with slope of $\frac{RT}{b}$ and an intercept of $\frac{RT}{b}lnK_{m}$ [253].

The comparison of the values of the fitting parameters including the correlation coefficient, obtained from the Langmuir, Freundlich, and Temkin models for several pairs are presented in Table 9.

## 9. Adsorption Kinetic Model

Adsorption kinetics also governs the rate of adsorption, which dictates the time necessary for the adsorption process to achieve equilibrium. Kinetic models may provide data on adsorption paths and on the mechanism likely involved. This is also essential for the developing and designing adsorption systems. Various kinetic models are available for identifying the intricate dynamics of the adsorption process. Kinetic studies have been conducted in batch trials using linear and/or non-linear regression equations to establish the best-fitting kinetic model. The best-fitting model is chosen depending on how close the correlation coefficient (*R^2^*) is to 1 [246]. The most common kinetic models are the pseudo-first-order kinetic model and the pseudo-second-order kinetic model, both of which help grasp the finer details of the adsorption process [247].

### 9.1. Pseudo-First-Order Kinetic Model

The pseudo-first-order model assumes that the rate of change in solute uptake with time is directly proportional to the difference in saturation concentration and the amount of solid uptake with time. This model is generally applicable during the initial phase of an adsorption process. When adsorption occurs by diffusion at the interface, it is widely observed that the kinetics follow this pseudo-first-order rate equation. Based on the adsorption capacity, the pseudo-first-order kinetic model is used to calculate the adsorption rate [258]. It is given by the equation below.

$$\log\left(q_{e}-q_{t}\right)=\log\left(q_{e}\right)-\left(\frac{k_{1}}{2.303}\right)*t$$

where *q_t_* and *q_e_* are the amount of solute adsorbed per mass of sorbent (mg g^−1^) at any time and equilibrium, respectively, and *k_1_* is the rate constant of first-order sorption (min^−1^). The straight-line plot of log (*q_e_ − q_t_*) against t gives log (*q_e_*) as the slope and an intercept equal to *k_1_*/2.303. Hence, the amount of solute sorbed per gram of sorbent at equilibrium (*q_e_*) and the first-order sorption rate constant (*k_1_*) can be evaluated from the slope and the intercept [259].

### 9.2. Pseudo-Second-Order Kinetic Model

The pseudo second-order kinetic model predicts behavior across the whole range of adsorption based on the premise that the rate-limiting phase is chemical sorption or chemisorption. This model has a significant benefit over Lagergren’s first-order model in that equilibrium adsorption capacity can be computed from the model; hence, there is theoretically no need to test the equilibrium adsorption capacity experimentally [247].

$$\frac{t}{q_{t}}=\frac{1}{k_{2}q_{e}^{2}}=\left(\frac{1}{q_{e}}\right)*t$$

where *k_2_* is the rate constant, and *q_t_* is the adsorbate uptake capacity at any time *t*.

External film diffusion, intraparticle diffusion and adsorption are fundamentally included in both pseudo-first-order and pseudo-second-order models [246]. The comparison of the values of the fitting parameters including the correlation coefficient, obtained from the pseudo-first-order and pseudo-second-order kinetics models for several pairs are presented in Table 10.

## 10. Mechanism of Enhancing Adsorption Performance

Activated carbon is a widely used effective adsorbent for removing a wide variety of pollutants dissolved in aqueous media or from a gaseous environment, owing to its exceptionally high porosity, well-developed internal microporosity structure and the presence of surface functional groups. Recent research indicates that modifying the surface of AC can enhance adsorption capacity. Because it is uncertain which nuclides are being adsorbed, we cannot change the adsorbates. However, the modification of the adsorbents (in this case, activated carbon) is possible; therefore, their adsorption capacity increases. Based on extensive literature reviews, it was found that there are three kinds of fundamental modification procedures. These are physical modifications, chemical modifications and organic or inorganic loading variations [262]. In the case of physical modifications, microwave heating, UV radiation, steam modification and other physical activation techniques are utilized to modify the surface of activated carbon. Modern researchers are investigating different methods to develop the surface properties of activated carbon by changing the pyrolysis temperature, heating time and heating rate, which may include UV and microwave radiation during the pyrolysis process [262]. Numerous activated carbons have been modified and functionalized to improve radionuclide adsorption capacity. Researchers have utilized strong acids, bases, polymers and other synthetic materials to change the functional groups and surface areas of activated carbon. As a result, activated carbon’s specific surface area and adsorption capacities are enhanced [263]. Various organic and inorganic substances can also be used to study the attachment of functional oxygen groups to carbons [264].

**Figure 10:**
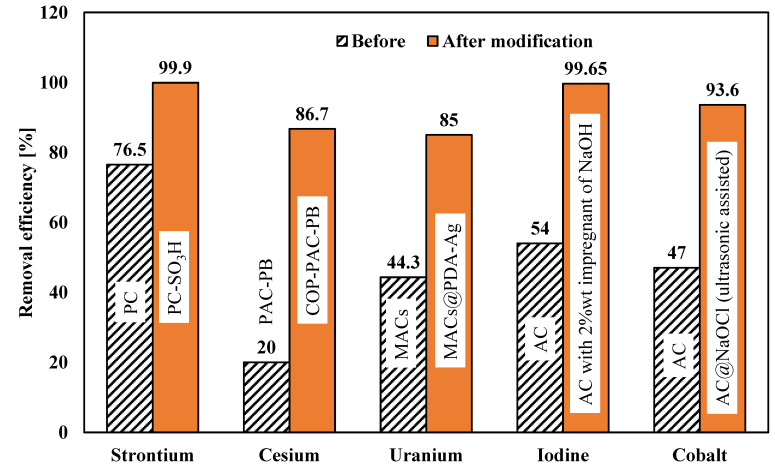
Comparison of radionuclide removal efficiencies before and after modification of parent activated carbon.

Different radionuclides need different types of functionalization of activated carbon to increase adsorption capacity or removal performance. Baik et al. [265] added sulfonic acid to CO_2_-derived porous carbon composites to increase strontium removal. According to this study, this technique enhances the adsorption capacity from 12.11 mg g^−1^ to 18.97 mg g^−1^. Nezhad et al. [227] initially used sulfuric acid to dissolve ammonium persulfate (APS) for uranium removal. Then, this mixture was used to add carboxyl groups to acetic acid, which was further functionalized using 2-aminobenzoic acid (ABA). Following this technique, the adsorption capacity improved from 24.5 mg g^−1^ to 48.5 mg g^−1^. In another study in 2021, Zhang et al. [266] used magnetic activated carbon functionalized by poly-dopamine (PDA) and Ag nanoparticles (AgNPs) to remove uranium from seawater. This technique increased removal efficiency from 44.3% to 85%. Kim et al. [267] investigated radioactive cesium. A Prussian-blue-impregnated adsorbent was developed by reforming the activated carbon surface with covalent organic polymers (COPs). Normal powdered activated carbon initially had a cesium removal rate of 20%. However, the cesium removal rate after surface modification went up to 86.7%. Babatunde et al. [236] used nitric acid and acetic acid to modify activated carbon derived from biomass precursors. Ordinary porous activated carbon has an adsorption capacity of 806.0 mg/g for iodine removal. However, the adsorption capacity increases to 1385.5 mg/g after nitric acid modification and 1284.5 mg/g after acetic acid modification. In another study of iodine-131 removal, Sadighzadeh et al. [106] used 2%wt NaOH to impregnate activated carbon. This increased the removal efficiency from 54% to 99.65%. Kakavandi et al. [237] studied the chemical modification of granular activated carbon (GAC) with sodium dodecyl sulfate (SDS), which yielded modified GAC (MGAC). It worked as an excellent adsorbent for removing Co(II) ions from aqueous solutions. According to this article, conventional porous GAC has an adsorption capacity of 16.4 mg/g for cobalt removal, but the adsorption capacity increases to 40.8 mg/g after surface modification. In 2011, Liu et al. [268] employed ultrasonic irradiation to modify activated carbon (AC) with sodium hypochlorite and then used this to remove Co(II) from aqueous solutions. This modification changed the removal efficiency from 47% to 93.6%. Figure 10 shows a comparison of the removal efficiencies of several radionuclides before and after the modification of parent materials. It can be seen that the removal efficiencies increase significantly after modification.

## 11. Conclusions

As nuclear power production has expanded in recent years, water contamination from radionuclides has become a more serious environmental issue that should be resolved to safeguard people and the environment from the adverse effects of radiation. This review aims to aggregate the literature on radionuclide removal from water employing activated-carbon-based adsorption techniques. It presents a comparative analysis of various removal techniques and the performances of many widely used adsorbents. This study indicates that adsorption techniques employing many adsorbent materials have more potential than other techniques. It also summarizes the key surface properties of widely used adsorbents for removing radionuclides from aqueous solutions. It was found that activated carbon is considered a superior material due to its specific structure, an abundance of functional groups and the possibility of carrying out additional functionalization. More importantly, it can be produced from abundant waste biomass sources, which are cost-effective and renewable. It discusses the synthesis processes of ACs from diverse biomass precursors and their radionuclide adsorption performance. It was observed that the nature of biomass precursors, activating agents, activation conditions and surface properties are crucial factors that can affect the adsorption capacity of AC. Furthermore, this review also focuses on modifying activated carbon’s properties to improve radionuclide adsorption performance. It was found that removal efficiency increases significantly after modification, and developing modified activated carbon for better removal could be an emerging research area. Adsorption with activated carbon prepared from biomass/biomass wastes and agricultural wastes/byproducts appears more viable than other radionuclide removal technologies based on its performance, adsorption capacities and cost. Despite substantial progress in the development of activated carbon and its application for radionuclide removal from wastewater, there is still an immense need for further research to find optimum synthesis conditions, optimum adsorption conditions and appropriate agents for modifying a particular radionuclide. It is recommended that experimental data and simulation studies should be employed to design a real adsorption system for removing radionuclides from wastewater. In general, this comprehensive review can significantly help researchers, academics and industrial scientists in selecting highly efficient, practical and environmentally compatible adsorbents for radionuclide removal from aqueous solutions. As a result, it can promote industries interested in producing waste-biomass-derived AC while preventing the production of harmful gases, such as CH_4_ and CO_2_, through the natural decomposition of biomass wastes.

## Figures and Tables

**Table 1:** Concerned radionuclides and their characteristics.

Radionuclide	Isotope Mass	Physical Half-Life	Biological Half-Life	Decay Mode	Energy of Decay Particles (MeV)	Health Hazard	References
Cesium-137	136.91 u	30.17 years	70 days	β^−^	0.514	Acute radiation sickness Burns Risk of cancer	[55,56,57]
Radioactive Iodine (I-131)	130.9 u	8.02 days	138 days	β^−^ and γ	0.606 for β^−^ 0.364 for γ	Burns to the eyes and skin Exposure to the thyroid gland	[34,35,36,58]
Zinc-65	64.9 u	243.8 days	933 days	β^−^ and γ	0.33 for β^−^ 1.116 for γ	Stomach cramp Skin irritation Anemia	[59,60,61]
Radon	222 u	3.8 days		A	0.024	Coughing up blood Chest pain Lung cancer	[62,63]
Strontium-90	89.9 u	28.8 years	18000 days	β^−^	0.546	Cancers in bone marrow Skin burns	[56,57,64]
Uranium	238.02 u	4.468 × 10^9^ years	15 days	A	4.267	Liver cancer Lung cancer due to over-inhalation Kidney damage	[56,61,65]
Cobalt-60	59.9 u	5.3 years	10	Γ	1.17 and1.33	Skin burns Acute radiation sickness	[56,61,66]

**Table 2:** Advantages and limitations of different removal techniques.

Removal Techniques	Advantages	Limitations	References
Adsorption	Easy operating conditions High efficiency (up to 99%) Cost-effective pH range is wide (2–12) High metal-binding capabilities, so most of the metals/nuclides can be removed.	Performance depends upon adsorbent Waste being produced In some cases, regeneration of the used adsorbents is necessary to resume the process.	[10,86]
Evaporation	Pure effluent	High operational cost (678.16 USD per month with 1.5-cubic-foot tanks) Generates sludges High energy consumption (25–50 kW per ton of evaporated water).	[12,87]
Ion exchange	Pure effluent Metal recovery	Not many nuclides can be removed High total cost (200,000 to 300,000 USD).	[12,13]
Electrodialysis	High selectivity for separation	The operating costs are high due to high energy consumption and membrane fouling (3900 USD for a capacity of 1 m^3^ h^−1^).	[10,88]
Membrane filtration	Less space needed High separation selectivity	High capital cost (11,564 USD for a capacity of 4 m^3^ h^−1^) High operational and maintenance costs for membrane fouling (964.51 USD for a capacity of 4 m^3^ h^−1^, which is 3.5 times higher than that of the electrodialysis method)	[10,88]
Chemical precipitation	Low cost (0.04 USD per gallon) Most nuclides can be removed	A large amount of sludge generation Extra sludge disposal cost	[10,11]

**Table 3:** Advantages and disadvantages of different adsorbents.

Adsorbent	Advantages	Disadvantages	References
Activated carbon	An efficient adsorbent with the highest capacity; produces high-quality treated effluent Relatively cheap Carbonaceous materials are available everywhere	Ineffective against dispersing and vat dyes	[90,91]
Zeolite	As the equipment is small, it takes up less space. In terms of maintenance and operation, it necessitates less expertise Cheap	Because it affects minerals, highly acidic water is not suited Regenerating iron and manganese zeolites is challenging Contaminants suspended in the raw material must be eliminated	[19]
Graphene	Water dispersibility is high Colloidal stability is good Large surface area (2629 m^2^ g^−1^)	Limited number of sorption sites Oxygen-containing functional group has a low density Expensive	[92,93]
Nanotube	Formation of high-stability bonds Simple procedure and minimum damage	Expensive Newer technology, so not as much testing has been completed	[94]
MOFs	High efficiency (up to 99.5%) Rapid synthesis Good porosity (<2 nm)	Long reaction time Byproducts are easily produced Expensive A large number of solvents are needed Still in the research and development phase	[20,21]

**Table 4:** Summary of experimental conditions, adsorption capacity and surface properties of different adsorbents.

Adsorbent Type	Adsorbent/ Adsorbate	Temperature (K)	Pressure	Concentration (mg L^−1^)	P_H_ Level	BET Surface Area (m^2^ g^−1^)	Pore Size (nm)	Pore Volume (cm^3^ g^−1^)	Removal Efficiency (%)	Adsorption Capacity (mg g^−1^)	Reference
**Activated Carbon (AC)**	BAC/Cs	288–308		20–800	2–12	347.72		0.1817	98%	55.25	[96]
	MAC/Cs	298–338		10–350	5.6					135.28	[100]
	Hexacyanoferrate-AC/Cs	298		1000	7	246	2	0.12		101.5	[101]
	AC/Sr	313		50	6	188	8	0.24	91.4%	50	[102]
	MMPC/Sr	298		1	10	667	2	0.62	93.3%	42.5	[103]
	PSBAC/Sr	343		100	6	1517		0.7	98%	8.12	[104]
	SACFP/iodine	383			8–10	950–1000			99%	850.5	[105]
	AC/iodine	328		169.069	10	499	2.2		98.5%	909.091	[106]
	SVAC/iodine	298		150	6.94	1178 (micro) 318 (meso)		0.4	99%	1178	[107]
	AC/uranium	303		83.72	5	364.17		0.15	92 ± 4%	50.539	[108]
	MRSAC/uranium	298		60	4				97.6%	11.36	[97]
	PAF/uranium	293		1	5	303	62		98.5%	115.31	[109]
	MCSG60A/cobalt	298		20	6	911	6	1.12	85–90%	1.5	[110]
	AC/cobalt	303		13.30–45.55	6	441			90%	13.88	[111]
	AC/cobalt	298–323		80	9		0.3–1.6		93%	111.11	[112]
**Zeolite**	MBS zeolite/cesium	298		100	2–12	103.631		0.28	97%	51.02	[113]
	nFe/Cu–Z/cesium	298		100	6	900	22		73.72%	77.51	[98]
	nZVI–Z/cesium	313		200	6	900	22		63.11%	71.12	[98]
	nFe/Cu–Z/strontium	343		100	12	900			89.73%	88.74	[114]
	nZVI–Z/strontium	343		100	12	900			86.82%	84.12	[114]
	Zeolite@Alg-Ca/strontium	328.15		140	4				96.48%	88.31	[115]
	Organo-modified zeolite/iodine	306		120	7.1–6.8				90%	4.02	[116]
	ZIFs/iodine	323		1000		897	1.87		85%	226	[117]
	Synthetic zeolite/iodine	298		375	2–12	51 ± 5.6	10 ± 0.1	0.13 ± 0.05	94.85%	20.44	[118]
	Clinoptilolite zeolite/uranium	298	<1	5	6	18	16.6	0.027	95.6%	0.7	[119]
	MOCZ/uranium	293	−3.5	25–400	4				91%	15.1	[120]
	HEU-type zeolite/uranium	298		100	4.5–7		200		88%	11.68	[121]
	Zeolite/cobalt	303–333		200	2–7.5				78%	120.9	[122]
	Sodium-modified Zeolite/cobalt	333		265	7.2				98%	20.73	[123]
	Zeolite/cobalt	298		50		50			98.7%	2.73	[124]
**Graphene**	Graphene oxide/cesium	283			9	93.7	2.43	0.31		95.46	[125]
	PB/Fe_3_O_4_/GO/cesium	298	1	100	7		15		80%	43.52	[126]
	PB-GO-Alg bead/cesium	273	1	10,000	5–7	130.2	146.1		98%	290.6	[127]
	GO/strontium	303		1000	6				91%	137.80	[128]
	GO/strontium	298		150	5	232	79.5	0.40	90%	131.4	[129]
	Polymer GO/strontium	298	1	5	6		450		99%	145.77	[130]
	GO/iodine	308		1.5	7.2 ± 0.2		200		92.6%	30.52	[131]
	Porous graphene/iodine	298	1	300	5–7	1755	2.5	1.31		4110	[132]
	Bi-GO/iodine	298	0.99 to 1.01	10	6.2	12.7			95%	200–230	[133]
	AMGO/uranium	328	1	42.84	5.9	59.09	200	0.37	90%	141.2	[134]
	rGO/uranium			100	4	162.92			94.76%	134.23	[135]
	rGO/LDH/uranium	298		130	4	256.80	4.53	0.66	99%	250.6	[135]
	GO-NH_2_/cobalt	298		300	6	320	220		90%	116.35	[136]
	β–CD/GO/cobalt	303	1	100	11				98.5%	72.4	[137]
	(GO)/chitosan/cobalt	293–323			5–9	27.15	1.953	0.127		15.24	[138]
**Nanotube**	Oxidized MWCNTs/cesium	298	0.0001–0.99	5–75	10	83.5	36	0.24	45%	12.75	[139]
	Amino-MWCNTs/cesium	308	0.99	14.79	7	112.5			95%	136.3	[140]
	PBA-CS-CNTs/cesium	293 ± 2		200	6				90%	219.8	[141]
	Oxidized MWCNTs/strontium	298	0.0001–0.99	5–20	6.5	83.5	40–60		85%	6.62	[99]
	CNTs/strontium	297 ± 2		3	3–9	500	8		95%	4.41	[142]
	MWCNTs/strontium	298 ± 2		6	6.5	88.53			97%	9.18	[143]
	SWCNTs/iodine	298	<1	0.05–50		560			95%	35	[144]
	SWCNTs/iodine	298		333		570			22.60%	1.356	[145]
	Ag-CNTs/iodine	298	0.01–0.3		8	72.14				458 ± 73	[146]
	PAO@CNT/uranium	298	0.993	56	4	58.169	40.88	0.595		247	[147]
	CS-CCN_2_/uranium	298	0.05–0.2	120	5	106.4	17.3		92%	307.5	[148]
	PVA/MWCNTs/uranium	298		100–1000	3	99	20–30		98.5%	232.55	[149]
	Magnetic MWCNTs/cobalt	333.15		4.2	6.3–6.5				96%	2.88	[150]
	MWCNTs/cobalt	293–313	10^-5^–0.998	56.57	10	370	3.8		90%	78.94	[151]
	NaAlg-HAp-CNT/cobalt	293		400	7.4	163.4	15–20			347.8	[152]
**Metal–Organic Framework (MOF)**	Nd-BTC MOF/cesium	308–338	0.99	1000	8	582	1.04	0.28	92%	86	[153]
	MOF-KNiFC/cesium	298–328		100	5	47.74	20.04	0.13		153	[154]
	MOF-Fe_3_O_4_-KNiFC/cesium	298–328		100	4	111.7	8.897	0.25		109	[154]
	Nd-BTC MOF/strontium	308–338	0.99	1000	8	582	1.04	0.28	78%	58	[153]
	ZnOx-MOF@MnO_2_/strontium	298		10–400	11	122.18	3.726	0.091	88.28%	147.094	[155]
	Fe_3_O_4_@UiO-66-NH_2_-MOF/strontium	298		0.05–5		878	8–10	0.69		0.4	[156]
	MIL-101(Cr)-SO_3_Ag/iodine	303	0.99	127–2305	7.5	861		0.4		244.2	[157]
	MIL-101(Cr)-SO_3_H/iodine	303	0.99	127–2305	7.5	1588		0.66		94.1	[157]
	Lac-Zn-MOF/iodine	298	0.1–1	50.80	6–7	227	15.71		92.89%	755	[158]
	Amidoxime MOF/uranium	298		9.8	9	1035	8		99%	2.68	[159]
	UiO-66-NH_2_/uranium	313.15	0.9–0.99	500–600	8	400	100–200	0.21	97.3–98.1%	278	[160]
	nZVI-UiO-66/uranium	313		80	6	1025.305	11.1	0.444	80%	404.9	[161]
	UiO-66-Schiff/cobalt	288–318	1	10–72.5	8.4	503	3.41	0.15		256	[162]
	Cr-MOF-AC/cobalt	298–318		70	5	2440	200	1.27		138	[163]
	Co(Ⅱ)-ⅡP-MOF/cobalt	288–308		10	8.4	482.46	3.41	0.189	90%	175	[164]

**Table 5:** Favorable weather conditions, annual production and availability of biomass precursors in Bangladesh.

Biomass Precursor	Favorable Weather	Annual Production in Bangladesh	Regions with Plentiful Growth	Reference
Rice	Hot, humid atmosphere	54.9 million tons	Almost every district of Bangladesh, especially in Rajshahi, Dinajpur, Dhaka, Chandpur, Mymensingh, Sylhet, etc.	[200]
Bamboo	Warm temperate, tropical regions	1500 pieces per hectare	Chittagong hill tracts	[201]
Sugarcane	Warm, humid atmosphere	3.68 million metric tons	Chittagong, Comilla, Bogra, Dinajpur Sylhet, Dhaka, Faridpur, Jamalpur, Jessore, Kushtia, Pabna, Rajshahi, Kishoreganj, Tangail and Rangpur.	[202]
Coconut	Relative humidity between 80–90% and annual rainfall of 1500 mm	431,596 tons	Coastal areas such as Chittagong, Teknaf, Khulna, Bagerhat, etc.	[203]
Potato	Rainfall of 400 to 600 mm, temperatures between 18 and 20 °C	9.61 million metric tons	Rangpur, Jessore, Meherpur, Thakurgaon, Dinajpur, Sherpur and Chuadanga.	[204]
Mangrove wood	Sufficient rainfall and a temperature range of 15–25 °C (not less than 10 °C)		Sundarbans	[205]

**Table 6:** Summary of activation conditions for the production of AC from different biomass precursors.

Precursors	Initial Carbon Content (%)	Widely Used Activating Agent	Activation Process Steps	References
Rice husk	30–50%	NaOH, KOH, HCL, Ozone	Physical activation in a muffin furnace for 1 h, temperatures ranging from 300 to 700 °C Weigh Soak in 1M KOH for 24 h Weigh again to know the impregnation of KOH activation in a muffle furnace at 300 °C for 2 h Wash with distilled water to remove free alkaline Dry at 100 °C for 2 h	[206,208]
Bamboo	39%	KOH, H_2_SO_4_, KMnO_4_, ZnCl_2_	**Chemical activation:** Using a muffle furnace, carbonize at 400 to 500 °C for 3 h Wash and dry at 110 °C for 6 h **Physio-chemical activation:** Carbonize dried bamboo in a muffle furnace at two temperatures of 400 °C and 500 °C for 3 h Impregnate with ZnCl_2_ Wash and dry at 100 °C for 6 h	[209,210]
Coconut shell	30–40%	KOH, Potassium acetate, H_3_PO_4_	**Chemical activation method:** Pyrolysis at 500 °C and 700 °C for 1 h Seal for 48 h Wash with distilled water Dry for 8 h at 100 °C	[209,211]
Potato peels/ Rotten potato	44%	KOH, ZnCl_2_, H_3_PO_4_, CO_2_	The activation is performed in two consecutive processes: **First, activation with H_3_PO_4_:** Mix potato peels, H_3_PO_4_ (85%) and water Stir for 2 h at 100 °C and dry for 24 h Pyrolyze at 500 °C for 30 min under a N_2_ atmosphere Cool and dry for 24 h at 120 °C. **Second, activation with KOH:** Mix KOH and stir at 100 °C for 1 h. Dry for 24 h at 120 °C Pyrolyze at 500 °C for 30 min in a N_2_ atmosphere Cool and wash with HCl Dry for 24 h at 120 °C.	[212,213]
Sugar-cane bagasse	35–45%	ZnCl_2_, H_3_PO_4_, Steam	Dry at 110 °C, cross for 15 min in a food processor Put inside the muffle furnace for 4 to 5 h at 800 °C and then cool The muffle furnace contains sand in two layers with sugarcane bagasse in the middle, surrounded by mud	[214,215]
Mangrove wood	47%	HCL, KOH	One hour of boiling in water at a temperature of 100 °C After filtration, dry the solid portion for 24 h at 105 °C Initiate carbonization process with gradual pyrolysis at temperatures between 300 °C and 325 °C for 1 h After that, completely immersed in KOH for 24 h Then, for one hour, carry out activation procedure in an electrical furnace set at 400 °C	[216,217]

**Table 7:** Surface properties of activated carbon derived from different biomass precursors.

Raw	Activating Agent	BET Surface Area (m^2^ g^−1^)	Pore Volume (cm^3^ g^−1^)	Pore Size (nm)	Reference
Rice husk	ZnCl_2_/CO_2_	480	1.3652	4.4	[27]
	NaOH	2681	1.4016	4	[175]
	KOH	2696	1.496	2.634	[176]
	FeCl_3_ solution	99.32	0.332		[219]
Bamboo	H_2_SO_4_	825.456	0.236	0.6077	[177]
	H_3_PO_4_	1492.50	0.731	2.49	[178]
	Microwave induced H_3_PO_4_	1432	0.696	-	[220]
Coconut shell	KOH	1118.2	0.4992	0.49	[221]
	HCL	525	0.291	1.42	[222]
	NaOH	346	0.199	1.256	[222]
Potato peels	KOH	833	0.44	2.23	[212]
	ZnCl_2_	1357	1.065	2	[22]
	ZnCl_2_	1078	0.97	<1	[213]
Sugarcane bagasse	H_3_PO_4_	661.46	0.2455	2.48	[223]
	ZnCl_2_	1502	0.886	0.85	[224]
	ZnCl_2_	1495	0.88	0.85	[217]
	CO_2_	906.1	0.174	1.505	[218]
Mangrove wood	KOH	2991	2.68	1.81	[34]
	H_3_PO_4_	2806	1.746	1–5	[225]
	H_3_PO_4_	561.6	0.26	1.126	[226]

**Table 8:** Adsorption capacities of radionuclides onto AC prepared from several potential biomass precursors.

Precursor	Radionuclide	Activating Agent	pH Level	Concentration (mg L^−1^)	Adsorption Capacity (mg g^−1^)	Reference
Rice husk	Cesium	Titanium silicates	6	70	13.58	[227]
	Strontium	Potassium hexacyano ferrate	6.89	100	42.5	[229]
	Iodine	NaOH, ZnCl_2,_ KOH		25–1000	1726	[230]
	Uranium	KOH	5.5	100	84.5	[231]
	Cobalt	Silica gel	8	100	75.7	[232]
Bamboo	Cesium	NaCl, KCl, NaOH, HCl	12	100	55.25	[96]
	Strontium	Al_2_O_3_, KOH	9.5	10	32.62	[28]
	Iodine	Steam	-	--	942	[233]
	Uranium	Amidoxime	7	100	396.51	[234]
	Cobalt	Sodium dodecyl sulfate	6	20	51	[235]
Coconut shells	Cesium	Physical activation	8.15	30	0.76	[31]
	Strontium	Physical activation	Alkaline	10.30	2.02	[31]
	Iodine	HNO_3_, KOH	-	-	1385.5	[236]
	Uranium	Physical activation	>5	100	55.32	[31]
	Cobalt	Na_2_SO_4_	6.87	5	0.09	[237]
Potato peels	Cesium	-	-	-	-	-
	Strontium	-	-	-	-	-
	Iodine	H_3_PO_4_	6.8	-	420	[30]
	Uranium	Ferrous sulphate	3	100	54.5	[238]
	Cobalt	H_3_PO_4_	6	200	405	[228]
Sugarcane bagasse	Cesium	Prussian Blue	10	300	56.7	[29]
	Strontium	NaOH	9	10–100	17.6	[239]
	Iodine	H_3_PO_4_	-	-	889.37	[240]
	Uranium	EDTA	5	100	1394.1	[241]
	Cobalt	Sulphurized AC	6	50 and 100	153.85	[242]
Mangrove wood	Cesium	Carboxyl, carbonyl and hydroxyl functional groups	7	50	133.54	[35]
	Strontium	-	-	-	-	Not available
	Iodine	H_3_PO_4_	7	-	1019.87	[243]
	Uranium	HCl	4	50	16	[244]
	Cobalt	-	6.9	10–50	3.18	[245]

**Table 9:** Summary of fitted adsorption isotherm model parameter’s value of several radionuclides.

Isotherm Model	Adsorbate	Adsorbent	Parameters			Reference
**Langmuir**			***q_m_* (mg/g)**	***K_L_* (L/mg)**	***R^2^***	
	Cesium	Oxidized BC	55.25	0.021	0.991	[96]
	Cesium	Nitric acid–modified BC	45.87	0.278	0.991	[254]
	Cesium	Modified molten slag	52.36	0.1496	0.989	[255]
	Strontium	Pristine biochar	41.2	0.0023	0.999	[256]
	Strontium	Magnetic biochar	40.2	0.0017	0.999	[256]
	Uranium	AC/PAN composite	27.47	0.031	0.949	[257]
	Cobalt	Potato peels AC (400 °C)	373	0.035	0.998	[228]
	Cobalt	Potato peels AC (600 °C)	405	0.050	0.995	[228]
**Freundlich**			***K_f_***	***(1/n)***	***R^2^***	
	Cesium	Oxidized BC	1.474 L/mg	0.535	0.966	[96]
	Cesium	Nitric acid–modified BC	10.56 mg/g	0.2628	0.797	[254]
	Cesium	Modified molten slag	12.16 L/mg	0.269	0.988	[255]
	Strontium	Pristine biochar	25.8 mg^1−(1/n)^ L^1/n^ g^−1^	0.244	0.949	[256]
	Strontium	Magnetic biochar	23.5 mg^1− (1/n)^ L^1/n^ g^−1^	0.268	0.950	[256]
	Uranium	AC/PAN composite	1.398 mg/g	0.598	0.883	[257]
	Cobalt	Potato peels AC (400 °C)	57.68 mg^1−(1/n)^ L^1/n^ g^−1^	0.302	0.937	[228]
	Cobalt	Potato peels AC (600 °C)	72.40 mg^1−(1/n)^ L^1/n^ g^−1^	0.285	0.947	[228]
**Temkin**			***b* (kJ/mol)**	***A* (L/g)**	***R^2^***	
	Cesium	Modified molten slag	0.26	0.81	0.995	[255]
	Uranium	AC/PAN composite	0.588	-	0.925	[257]

**Table 10:** Summary of fitted adsorption kinetics model parameter’s value of several radionuclides.

Adsorbate	Adsorbent	Concentration (mg L^−1^)	*k_1_* (min^−1^)	*k_2_* (g/mg.min)	Calculated *q_e_* (mg g^−1^)	Experimental *q_e_* (mg g^−1^)	*R^2^*	Model Name	Reference
Cesium	Bamboo-based AC	20–800	-	0.314	9.090	9.021	0.999	Pseudo-second-order	[254]
	Oxidized bamboo-based AC	20–1000	-	0.016	3.44	3.16	0.998	Pseudo-second-order	[96]
	Modified molten sludge	20–800	-	1.37	10.30	10.30	1.0	Pseudo-second-order	[255]
Strontium	Pecan shell AC	25–100	-	0.008	6.17	-	0.98	Pseudo-second-order	[104]
	Magnetic SCG biochars	5–50	-	0.307	34.7	34.5	1.0	Pseudo-second-order	[256]
	Pristine SCG biochars	5–50	-	0.290	35.1	34.9	1.0	Pseudo-second-order	[256]
Uranium	Granular AC	100	-	0.004	25.51	24.44	0.99	Pseudo-second-order	[260]
	Granular AC	300	0.009	-	13.40	12.94	0.98	Pseudo-first-order	[260]
	AC/PAN	10–400	-	0.029	-	9.36	1.0	Pseudo-second-order	[257]
Cobalt	Doum stone AC	100–250	0.027	-	9.90	14.81	0.997	Pseudo-first-order	[261]
	Doum stone AC	100–250	-	0.073	15.93	14.81	0.999	Pseudo-second-order	[261]
	Potato peels AC	10–1000	0.0344	-	184.79	-	0.997	Pseudo-first-order	[228]

## Data Availability

The data are contained within this article or in the cited literature, accordingly.
